# Factors Associated with Utilization of Teleretinal Imaging in a Hospital-Based Primary Care Setting

**DOI:** 10.3390/vision7030053

**Published:** 2023-08-04

**Authors:** Kira J. Szulborski, Selin Gumustop, Claudia C. Lasalle, Kate Hughes, Shiyoung Roh, David J. Ramsey

**Affiliations:** 1 Division of Ophthalmology, Department of Surgery, Lahey Hospital & Medical Center, 1 Essex Center Drive, Peabody, MA 01960, USA; 2 Department of Ophthalmology, Tufts University School of Medicine, Boston, MA 02111, USA

**Keywords:** diabetic retinopathy, diabetes mellitus, screening, telemedicine, teleretinal imaging, cost analysis, integrated delivery network, quality improvement

## Abstract

Regular eye examinations to screen for the initial signs of diabetic retinopathy (DR) are crucial for preventing vision loss. Teleretinal imaging (TRI) offered in a primary care setting provides a means to improve adherence to DR screening, particularly for patients who face challenges in visiting eye care providers regularly. The present study evaluates the utilization of TRI to screen for DR in an outpatient, hospital-based primary care clinic. Patients with diabetes mellitus (DM) but without DR were eligible for point-of-care screening facilitated by their primary care provider, utilizing a non-mydriatic, handheld fundus camera. Patient demographics and clinical characteristics were extracted from the electronic medical record. Patients who underwent TRI were more likely to be male, non-White, and have up-to-date monitoring and treatment measures, including hemoglobin A1c (HbA1c), microalbumin, and low-density lipoprotein (LDL) levels, in accordance with Healthcare Effectiveness Data and Information Set (HEDIS) guidelines. Our findings demonstrate that TRI can reduce screening costs compared to a strategy where all patients are referred for in-person eye examinations. A net present value (NPV) analysis indicates that a screening site reaches the break-even point of operation within one year if an average of two patients are screened per workday.

## 1. Introduction

Diabetes mellitus (DM) is a global epidemic, impacting more than 537 million individuals worldwide, with projections estimating a rise to 783 million by 2045 [1]. Diabetic retinopathy (DR) is a prevalent microvascular complication of diabetes that affects approximately one-third of individuals with DM [2]. The development of DR is attributed to progressive damage to the retinal blood vessels caused by hyperglycemia and other diabetes-related factors [2,3]. Timely detection and treatment of DR are crucial in preventing vision loss [4]. While regular eye screenings to detect early signs of DR are recommended for all individuals with DM [5,6], adherence to these guidelines has been suboptimal [7]. A significant proportion of patients with DM do not undergo yearly eye examinations, even when they have health insurance coverage [8,9,10,11]. In certain U.S. populations, the rate of annual diabetic eye screening can be as low as 30%, in part because the patient and their physician have discretion over the timing and location of diabetic eye screening [12,13]. In contrast, countries with national health systems that have established universal DR screening programs have achieved considerably higher screening rates among eligible patients and have successfully reduced the incidence of blindness associated with DR [14,15]. The introduction of point-of-care teleretinal imaging (TRI) performed with non-mydriatic fundus cameras offers a cost-effective approach to enhance adherence to screening for DR [16,17,18,19,20,21,22,23,24,25,26,27,28,29,30]. This service is typically covered by most insurance plans [31] and is often tied to incentives aimed at achieving performance metrics in risk-sharing health insurance contracts [32].

We conducted a cross-sectional study to assess the utilization of TRI to screen for vision-threatening DR in an outpatient, hospital-based primary care clinic within an integrated delivery network (IDN). We compared the characteristics of patients who were referred for and successfully completed TRI with patients who were expected to continue traditional, in-person eye screening. We also assessed whether TRI could reduce the screening costs for low-risk patients without prior DR compared to a strategy of referring all eligible patients to undergo in-person eye examinations.

## 2. Materials and Methods

### 2.1. Study Design

In an outpatient, hospital-based general internal medicine clinic, patients with DM who had not been previously diagnosed with DR and were due for an annual diabetic eye examination were eligible for point-of-care remote retinal screening. Primary care providers (PCPs) were recommended to refer all such patients TRI and were regularly notified of the availability of the service through weekly communications, which were further reinforced during daily staff team meetings. Those patients referred for point-of-care remote retinal screening by their PCPs were compared to patients who did not undergo successful screening in the TRI program. TRI was performed asynchronously during the normal operation of the clinic. All images were obtained by trained medical assistants by using a handheld, non-mydriatic fundus camera with a 40-degree field of view (Visuscout 100, Carl Zeiss Meditec, Inc., Dublin, CA, USA) under direct supervision of PCPs from 2 June to 22 December 2021. Color fundus photographs centered on the disc and macula were acquired from each eye without pupil dilation and transmitted for evaluation, as previously described [29]. The interpretation of imaging studies was performed asynchronously, and the outcomes communicated to the PCP through a report in the electronic medical record. A board-certified ophthalmologist with expertise in retina (DJR) evaluated each image on a high-resolution, color-calibrated monitor. An image was considered ungradable if the photographic quality or media opacity made it impossible to determine whether DR lesions were present [13,17]. Patients with photographic evidence of DR or other potentially serious eye conditions were referred by their PCP to an ophthalmologist for further evaluation. By contrast, patients who showed no signs of DR received reports that informed them of their screening results, reminded them of the need to continue annual diabetic eye examinations, and advised them to seek in-person eye care sooner for any vision-related issues.

Patients were identified based on International Classification of Diseases, Tenth Revision, Clinical Modification (ICD-10-CM) codes for type 1 and type 2 DM without DR. Excluded were patients who died or transferred care during the study period. A customized electronic medical record reporting tool (EPIC Systems Inc., Verona, WI, USA) [33] was used to extract information regarding patient demographic and sociomedical characteristics (age, sex, self-reported race and ethnicity, primary language spoken, type of health insurance, tobacco use, and zip code), as well as clinical characteristics (type of DM and any associated complications, e.g., history of nephropathy or neuropathy, dates of completed primary care appointments, and relevant biometric data). Specifically, elements of the Comprehensive Diabetes Care bundle [34] (hemoglobin A1c [HbA1c] testing, HbA1c control [considered <8.0%], HbA1c poor control [considered <9.0%], eye exam [retinal] performed, medical attention for nephropathy, and blood pressure [BP] control [considered <140/90 mmHg]) as well as relevant Healthcare Effectiveness Data and Information Set (HEDIS) measures (low-density lipoprotein [LDL] testing, LDL control [considered <100 mg/dL], and body mass index [BMI]) were obtained. Mean household income per zip code was estimated using 2018 U.S. Census Data, dividing the total income by the number of tax returns for each respective zip code [35]. The approximate distance to the clinic for each patient was computed by using an Excel VBA program to access Microsoft Maps, which calculated the number of miles between each patient’s home and the clinic by zip code, as previously described [29]. Finally, in order to understand why some patients seen in primary care may not have been referred for TRI, those patients who completed imaging were matched 1:1 with those patients who did not utilize the service. The extent to which diabetic eye screening was addressed and the type of visit (i.e., a problem-focused visit versus routine care) were assessed through a manual review of each chart. The research followed the tenets of the Declaration of Helsinki and was approved by the Institutional Review Board of the Lahey Hospital & Medical Center, Burlington, MA, USA. Information was collected and secured in accordance with the Health Insurance Portability and Accountability Act.

### 2.2. Time-Driven Activity-Based Costing

Time-driven activity-based costing (TDABC) was performed, as previously described [29]. Briefly, the labor cost of a full-time medical assistant was calculated based on a schedule of 40 h per week, with five weeks off, for a total of 1880 h worked over the course of a year. The average salary for each team member was determined by referencing published data [36,37,38] and subsequently divided by the total number of work hours to calculate the corresponding hourly rate. The time required for an in-person diabetic examination was estimated to be 15 min (0.25 h) based on the eye clinic schedule. The time to complete remote retinal imaging averaged 12 min (0.2 h) and was calculated by recording the time spent with a patient from start of TRI to the completion of image transmission. Grading of retinal images was estimated to require approximately one minute per eye by an experienced grader (0.03 h) [29]. Our model also factored in the initial training cost for an ophthalmic technician ($23/h) and computer analyst ($27/h) to provide in-service to the medical assistants ($18/h) [38] on how to perform non-mydriatic fundus photography and use of the camera’s software, respectively. We additionally considered recurring costs from biannual trainings required for the proper use of a portable fundus camera ($182/year), as well as annual battery replacement ($100/year). The sum of these costs was $282 annually, or the equivalent of $23.50 per month.

### 2.3. Net-Present Value Analysis

A financial model created in Microsoft Excel utilizing the net-present value analysis (NPV) approach was used to calculate the profitability of an investment in a teleophthalmology site. The calculations for NPV utilized the equation [39]:

(1) $$\sum_{t=0}^{n}\frac{C_{F}}{{\left(1+r\right)}^{t}}-[cost\;of\;investment],$$

which represents the summation of all future cash flow (C_F_) from an investment minus the initial cost of that investment. In our pro forma model, the cost of a handheld non-mydriatic fundus camera was estimated to be approximately USD 10,000. For our NPV analysis, we employ a discount rate (r) of 3%, a rate frequently used in health-related economic evaluations and recommended in federal guidance on cost-effectiveness analysis of health interventions [40]. This discount rate was divided by 12 to arrive at a monthly interest rate of 0.25%. To calculate the projected monthly cash flows, we used a reimbursement rate of USD 33.93 for remote retinal screening, which is the 2021 allowable charge using current procedural terminology (CPT® 92228) [41], reduced by the ungradable image rate (approximately 12.1%). Potential savings derived from the differential costs of in-person eye examinations compared with teleophthalmology screenings were ignored in our model because of the uncertainty that all patients screened would have followed through with in-person eye care. The return on investment (ROI), i.e., monetary benefits of setting up a screening program site compared with the cost of the project, was calculated using the following equation [39]:

(2) $$ROI\left(\%\right)=\frac{C_{F}-[cost\;of\;investment]}{\left[cost\;of\;investment\right]}.$$

The useful life of a camera was set at five years, in part because newer technology would lead to its replacement. Cash flow (net and cumulative), internal rate of return (IRR), and payback period were calculated in Microsoft Excel utilizing this pro forma model.

### 2.4. Statistical Analysis

Data were analyzed using SPSS (version 28.0, IBM, Armonk, NY, USA). The chi-square test or Fisher’s exact test was utilized to assess the difference between categorical variables. Continuous variables were recorded as mean ± standard deviation (SD) and were analyzed using the Student’s *t*-test for normally distributed variables and Mann–Whitney U test for non-normally distributed variables as determined by the Shapiro–Wilk test. Binary logistic regression analysis was used to identify demographic, clinical, and sociomedical factors associated with TRI status. Odds ratios (ORs) and 95% confidence intervals (CIs) were calculated for each variable. All tests were two-sided, and *p*-values below 0.05 were deemed statistically significant.

## 3. Results

Of the 4743 patients with DM without previously-documented DR, 275 patients (5.8%) successfully completed screening for DR by means of point-of-care TRI. The average time spent with each patient was less than 10 min (8.4 ± 5.2 min). A further 38 patients had incomplete studies because of ungradable photographs (12.1%), requiring referral for an in-person eye examination. Finally, 18 patients with orders placed for TRI left the clinic without completing any imaging.

### 3.1. Demographic, Biometric, and Socioeconomic Factors Predicting Remote Screening

The demographics and clinical characteristics of the 275 participants who successfully completed TRI are summarized in Table 1.

The mean age of patients who received TRI was 65.7 ± 13.0 years compared with 66.9 ± 13.8 years for patients who did not undergo remote screening (*p* = 0.158). A greater proportion of patients were male in the TRI group compared with the patients who were not screened (71.3% versus 57.3%, *p* < 0.001). Patients who received TRI were less likely to identify as White and non-Hispanic (76.7% versus 82.8%, *p* = 0.01). Individuals who identified as members of other races and/or ethnicities were screened at rates proportional to their representation in the study population. Finally, none of the other demographic or sociomedical factors were associated with increased utilization of TRI, including type of DM, class of health insurance, smoking status, distance from the clinic, or estimated household income.

Several notable differences were identified in the biometric measures related to DM monitoring and treatment when patients who completed TRI were compared with those who did not access the program. A smaller percentage of patients who completed TRI had HbA1c measurements classified as controlled below a target of 8.0% than those who did not participate (76.6% versus 82.7%, *p* = 0.01). However, patients who were imaged were equally likely to have HbA1c > 9.0%, indicating poor control (10.5% versus 9.3%, *p* = 0.479). Within the year of the study, patients who were screened in the TRI program were also more likely to have completed an HbA1c measurement (97.8% versus 92.0%, *p* < 0.001), microalbumin testing (86.6% versus 64.6%, *p* < 0.001), and LDL testing (79.3% versus 69.1%, *p* < 0.001). However, patients were similarly likely to achieve a goal BP below 140/90 mmHg (73.8% versus 71.2%, *p* = 0.349), LDL less than 100 mg/dL (70.1% versus 71.0%, *p* = 0.750), or have a BMI below the range indicating obesity (BMI < 30 kg/m^2^; 47.3% versus 46.3%, *p* = 0.754). Finally, the rate of other diabetic complications was similar for those patients who received TRI compared with those who did not access the program (nephropathy: 13.8% versus 12.5%, *p* = 0.518; peripheral neuropathy: 36.0% versus 38.3%, *p* = 0.444).

To understand the factors contributing to the lack of TRI referrals, the study team conducted a chart review of eligible patients who were not referred, matching them 1:1 with patients who completed imaging. The review revealed that the majority of visits were routine follow-up examinations; fewer than 5.8% were categorized as problem-focused visits based on the documented chief complaint. Among the patients who were not referred for TRI, fewer than half had diabetic eye screening mentioned in the assessment and plan (41%). By contrast, 79% of visits had the other elements of the Comprehensive Diabetes Care bundle specifically addressed by the PCP in the note. Not surprisingly, when a PCP specifically attended to the other elements of the Comprehensive Diabetes Care bundle, there was a greater chance that the need for a diabetic eye examination was recognized and discussed with the patient (OR 10.400; 95% CI 4.001–27.035, *p* < 0.001). Finally, nearly two-thirds of the charts reviewed for patients who did not undergo TRI indicated that the patient had an existing plan for in-person diabetic eye screening. Not a single chart reviewed documented a specific reason why TRI was deferred.

### 3.2. Multivariate Regression Analysis for Factors Associated with Completing TRI

Multiple regression was used to assess the interaction of factors that were found to have a significant association with the completion of TRI (Table 2).

Variables included in the final model included male sex, White race, HbA1c < 8.0%, and completion of HbA1c, microalbumin, and LDL testing. Other values were not included in this model because of the absence of a unique predictive ability. Medical attention for nephropathy, as indicated by microalbumin testing, a part of the Comprehensive Diabetes Care bundle, was found to be the most important factor after controlling for the other variables in the model (OR 2.945; 95% CI 2.039–4.253, *p* < 0.001).

### 3.3. Detection of Diabetic Retinopathy

Eight patients (2.9%) were newly diagnosed with more than mild DR because of remote screening using handheld, point-of-care cameras in the TRI program. This is similar to the rate of newly diagnosed DR recorded among all patients with DM seen in the hospital outpatient primary care clinic during the year of the study (1.7% *p* = 0.116). Advanced age was modestly associated with the likelihood of being diagnosed with DR (OR 1.069; 95% CI 1.002–1.140, *p* = 0.043). No other biometric or sociomedical factors were significantly associated with newly diagnosed DR.

Although the effects of the coronavirus disease 2019 (COVID-19) pandemic may have caused some patients to defer eye care [42], one-third of patients who underwent TRI completed a subsequent in-person eye examination (92 patients). Three of these follow-up examinations were positive for DR (3.3%), including one patient referred because DR had been detected on TRI; the other two cases had no positive findings on TRI but were diagnosed with mild DR without macular edema by an ophthalmologist. By comparison, out of the remaining 1285 patients who did not undergo TRI but completed in-person eye examinations in the year after the study, approximately one-third of the total (77 patients) were newly diagnosed with DR (6.0%, *p* = 0.330). No biometric or sociomedical factors were significantly associated with newly diagnosed DR. Finally, it is worth noting that 16% of the patients in our study who completed TRI were newly diagnosed with DM.

### 3.4. Cost Analysis

Using TDABC, we determined that the cost associated with in-person dilated eye exams is approximately USD 41.53 for ophthalmologists (USD 166/h) and USD 17.64 for optometrists (USD 71/h). By contrast, acquiring images via remote retinal imaging incurs a cost of only USD 3.40 for the labor of the medical assistant and USD 4.98 for a physician to grade the images. Taking into account the 12.1% rate of ungradable imaging studies, this resulted in cost savings ranging from USD 4 to USD 28 per patient screened by TRI when compared to standard dilated retinal examinations performed by optometrists or ophthalmologists, respectively (Table 3).

**Figure 1:**
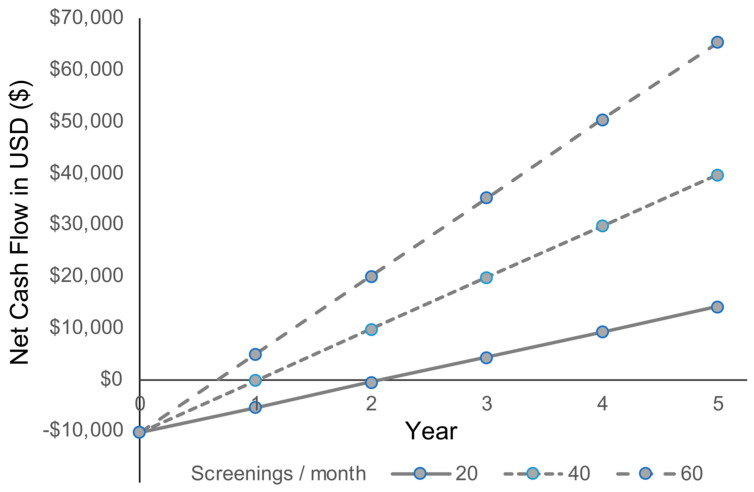
Projected nominal cash flow from the operation of a TRI program site. The graph illustrates the cumulative net cash flow for a TRI screening program site, presenting the relationship between the number of remote screenings performed per month and the corresponding financial outcomes. The break-even point for each scenario, where net cash flow reaches zero, is highlighted. This break-even point represents the threshold at which operational costs are covered before achieving profitability.

A sensitivity analysis using NPV was employed to determine how the number of remote screenings performed each month would impact the profitability of a TRI program site. The imaging program screened an average of 39.3 ± 9.5 patients per month (ranging from 32 to 55 patients). If 20 remote screenings captured per month were set as a low end in our model (i.e., one screening per workday), it would take just over 25 months to break even from the costs of investment (Figure 1; Supplemental Table S1). If, on the other hand, we set a higher average of 40 remote screenings per month (i.e., fewer than two per workday), it would take approximately 12 months to break even. Finally, in a best-case scenario where 60 photos were taken per month (approximately 2.8 screenings per workday), the break-even point would occur in less than nine months of operation. The results of a five-year cost-benefit analysis demonstrate that a site with such a volume is profitable based on the ROI (366%), IRR (115%), and NPV (USD 47,425).

## 4. Discussion

This study examines the use of TRI as a screening tool for DR in a low-risk, well-insured suburban population and reports on the cost of implementing such a service. Several factors were identified as predictive of program participation, including male sex, identifying with a race other than White, and having closer monitoring of elements in the Comprehensive Diabetes Care bundle, including HbA1c and microalbumin testing, and the closely aligned HEDIS metric, LDL testing. This is important because the progression of DR is closely linked to the control of DM, as reflected by HbA1c levels, comorbid nephropathy, and control of hypertension and hyperlipidemia [43,44,45].

Previous studies have revealed that certain demographic factors, such as sex and diabetes control, can influence the likelihood of male patients seeking in-person ophthalmology services, including during the COVID-19 pandemic [46]. Our study found that patients who received TRI were more likely to be male. Knowledge of this association may have prompted some PCPs to refer more male patients for imaging. Additionally, there might be an unconscious bias suggesting that male patients may be more open to adopting technology-based solutions [47], such as undergoing non-mydriatic fundus (retinal) photography. However, it is difficult to determine with any degree of certainty whether these factors directly contributed to the higher participation rate of male patients in the TRI program.

Implementation of TRI as a point-of-care test where patients with DM obtain primary care may provide a means to reach underserved patients, including those who identify as members of a racial or ethnic minority group. Those patients often have more difficulty accessing eye care [22,23], a problem further exacerbated by the COVID-19 pandemic [29]. In our study, we found that patients who identified as non-White were slightly more likely to access TRI compared with patients who identified as White. Importantly, both groups were equally likely to have had a prior eye examination in our well-insured suburban population (data not shown). As our suburban practice is relatively less diverse than many healthcare settings in the United States, we are unable to comment on the tendency of any individual racial or ethnic minority group to access TRI more than any other. Future studies should specifically address gaps in eye screening for patients who identify as members of racial or ethnic minority groups since individuals of those groups experience a disproportionate burden from DR and are historically less likely to access eye care [18,19,20,22,23,24,25,26,27,28,30].

Because of its efficiency and accuracy in monitoring and screening for eye disease [16,17,48,49], TRI is now recognized as one of the leading tools for population-wide DR screening by the World Health Organization and the American Academy of Ophthalmology [6,50]. The handheld camera used to screen patients for DR in our study has a reported sensitivity of 88% to 92% and a specificity ranging from 95% to 96% [17]. This remarkable specificity, coupled with its cost-effectiveness, positions TRI as a highly effective screening method for patient populations, like ours, that are expected to have a low prevalence of DR due to being previously determined as free of DR on a recent in-person eye examination [51] or having a relatively short duration of DM [44]. Interestingly, we found that patients who underwent TRI were slightly more likely to have completed a prior eye examination compared with those who did not access TRI (61% versus 54%, *p* = 0.03). However, it should be noted that the majority of those visits occurred prior to the onset of the COVID-19 pandemic. Nevertheless, even with extended intervals between in-person appointments, such lower-risk patients have been shown to have very infrequent progression to DR [51]. This finding is further supported by the very low rate of DR detected in one-third of patients who completed in-person eye examinations in the year after undergoing TRI in our study. Other studies also found a positive association between participating in a photographic screening program and subsequent adherence to receiving recommended in-person diabetic eye examinations [52,53]. One of the strengths of our study is that the results of in-person eye examinations indicating that the patients were free of DR were available in the electronic medical record within a few years of TRI for more than half of the patients in our study. This indicated that the risk of undiagnosed DR was relatively low for those patients. It is noteworthy that the rate of DR detected in our study population (3.3%) aligns closely with the expected yearly incidence of DR among patients with DM in the United States (3.6%) [54]. Importantly, none of the patients for whom we have subsequent in-person eye examination data were identified to have vision-threatening DR that had been overlooked on screening photographs. 

Our study revealed a strong association between the completion of a retinal examination using TRI and the comprehensive management of other Comprehensive Diabetes Care HEDIS metrics [34]. This suggests that significant effort is being put into completing these goals that are aimed at delivering effective DM care. Our health system offers patients the flexibility to choose between point-of-care TRI and traditional in-person DR screening, allowing them to select their preferred method and setting for receiving eye care. This may have the added advantages of reducing costs and increasing access and performing as well, if not better, in quality-adjusted life-years (QALYs) gained [30]. The anticipated cost savings generated by our TRI program within our not-for-profit IDN aligns with projections made by other studies that have examined the cost-effectiveness of TRI for DR screening [19,28,29,30]. Our cost analysis does not fully take into account the time required for program design, staff training, or other indirect expenses. However, these are one-time or fixed expenditures that would amortize during the ongoing operation of an imaging program.

In the U.S. healthcare system, a prevailing approach involves selective screening for DR, wherein patients and their physicians exercise autonomy in determining the timing, location, and extent of screening, including the adoption of technological solutions. Although providers were asked to refer all eligible patients requiring a diabetic eye examination for TRI, actual referrals were dependent on individual clinical judgment and follow-through. This contrasts sharply with the approach to DR screening in countries with national health systems that provide universal DR screening programs [14,15]. Practice managers frequently attributed the relatively low number of patients imaged to limited staff availability [29]. The data available to us are not sufficient to determine which patients were offered TRI or to identify the reasons why certain patients may have declined screening when offered. In the future, a formal assessment of the reasons why patients and their providers chose not to utilize TRI should be undertaken. Such an assessment may reveal opportunities for designing specific interventions to lessen barriers to care, such as those related to unfamiliarity with the technology and its ability to screen effectively or knowledge about coverage for the service.

TRI programs in the U.S. are predominantly implemented in specific settings such as the Veterans Affairs (VA) Health System, the Indian Health Service, and large county health systems [9,11,25]. These programs have provided valuable evidence of the effectiveness of TRI but may not be representative of the feasibility of its wider adoption in U.S. health systems because of differences in financial structure and electronic health record integration [15,28]. Furthermore, DR screening programs that rely on grant support often face challenges in achieving long-term financial sustainability after the end of funding. Our study highlights the potential for increasing insurance coverage for DR screening through TRI to promote the adoption of this approach by a wider range of healthcare organizations in the United States [31]. Furthermore, incentives for achieving performance metrics in risk-sharing health insurance contracts should further motivate the implementation of TRI [32,55].

The cost savings associated with remote diabetic screening tests compared to in-person eye exams may initially seem modest, with TRI screening saving between 23% to 67% per patient compared to in-person exams. However, this analysis fails to consider the additional expenses and inconveniences faced by patients who have to schedule and attend a separate appointment at an eye clinic, see a different provider, and potentially pay a co-payment, even if the exam is covered by insurance. TRI took less than ten minutes per patient and, as a screening test, did not require a separate co-payment. It is also important to take into consideration that conducting TRI as a point-of-care screening test during a primary care visit guarantees that the results are readily available to the PCP responsible for coordinating diabetes care. Our program also did not offer dilation of the eyes in cases where the images obtained were of poor quality. Fortunately, the impact of dilation on the gradability of images is modest [17], and many patients who have poor-quality media may benefit from in-person evaluation [13]. Although we cannot determine the long-term impact of this one-time intervention on patient adherence to follow-up recommendations, we believe that the value to the healthcare organization through other downstream care pathways is likely to be substantial.

This study is limited to the retrospective evaluation of the utilization of TRI in a well-insured, suburban population of patients served by a single academic hospital-based, primary-care outpatient clinic. In our study, our intervention was not randomized. We also did not control for the duration of DM, the complexity of its management, or other comorbid eye diseases, all of which may have influenced patient selection for remote retinal screening [44]. For example, relatively few patients with type 1 DM were included in our study. A possible reason is that patients with type 1 DM may be more likely managed by endocrinologists in our IDN. The inclusion of only those patients who had completed a primary care visit within the study period, which spans the COVID-19 pandemic, also fails to capture patients who may have been disconnected from the healthcare system. Notably, such patients face not only poorer DM control but also an increased risk of developing DR [44,46]. Our electronic medical record-based study also misses the outcomes of eye care visits that occurred outside of our health system and were not reported back to a PCP. Finally, our cost analysis does not take into consideration other long-term benefits of remote diabetic retinal screening, such as the earlier identification of patients at risk of severe vision loss [19,28,30], improved patient satisfaction brought about by access to point-of-care testing [56], and the potential for better performance in meeting HEDIS measures of health system quality [32].

## 5. Conclusions

Our study conducted among a suburban, well-insured population of patients with DM found that TRI was most often used to screen patients who were at a greater risk of not following through with traditional referrals for eye examinations, such as those who were male, identified as non-White, or had poorer metabolic control [7,22,23,46]. TRI provides an opportunity to reduce the costs of screening patients without prior DR compared with referring all eligible patients with DM for in-person eye examinations. TRI may also produce the best results for patients who have up-to-date measures related to DM treatment and engagement. The screening of as few as two patients per workday, a feasible target in a high-capacity, hospital-based primary care setting, can lead to cost neutrality within one year of operation. As the demand for eye care continues to outpace availability, TRI represents a promising strategy for delivering high-quality, accessible care in a cost-conscious way. Additional efforts are needed to raise awareness about the availability and benefits of retinal imaging for DR screening. Furthermore, the integration of teleophthalmology and artificial intelligence services should be considered to improve the effectiveness of screening programs.

## Figures and Tables

**Table 1:** Demographic and clinical characteristics overall and within those who participated in TRI and those who did not complete TRI. HbA1c, hemoglobin A1c; BP, blood pressure; LDL, low-density lipoprotein; BMI, body mass index.

Parameter	All Patients	Teleretinal Imaging		*p*-Value
		Completed	Not Imaged	
*n*	4743	275	4468	
Age (years)				
Mean (SD)	66.8 (13.8)	65.7 (13.0)	66.9 (13.8)	0.158
Median	68	66	68	
Range	18–100	22–97	18–100	
Male, *n* (%)	2756 (58.1)	196 (71.3)	2560 (57.3)	<0.001
Race, *n* (%)				
White, Non-Hispanic	3911 (82.5)	211 (76.7)	3700 (82.8)	0.010
Asian or Asian American	441 (9.3)	32 (11.6)	409 (9.2)	0.168
Black or African American	161 (3.4)	15 (5.5)	146 (3.3)	0.053
Hispanic or Latin American	43 (0.9)	2 (0.7)	41 (0.9)	0.747
More than One Race	26 (0.6)	1 (0.4)	25 (0.6)	0.663
Other Races or Ethnicities	125 (2.6)	12 (4.4)	113 (2.5)	0.066
Missing	36 (0.8)	2 (0.7)	34 (0.8)	0.956
Insurance Type, *n* (%)				
Commercial	1767 (37.3)	108 (39.3)	1659 (37.1)	0.476
Medicare	2511 (59.9)	134 (48.7)	2377 (53.2)	0.150
Medicaid	437 (9.2)	30 (10.9)	407 (9.1)	0.317
Other ^†^	28 (0.6)	3 (1.1)	25 (0.6)	0.266
Distance to Clinic (mi)				
Mean (SD)	18.71 (25.85)	20.48 (28.02)	18.60 (25.71)	0.651
Income by Zip Code ($K)				
Mean (SD)	105.7 (61.2)	104.3 (53.5)	105.8 (61.6)	0.851
Smoking Status, *n* (%)				
Never	2299 (48.5)	140 (50.9)	2159 (48.3)	0.404
Former	1908 (40.2)	105 (38.2)	1803 (40.4)	0.476
Passive	16 (0.3)	0 (0)	16 (0.4)	0.319
Current	456 (9.6)	27 (9.8)	429 (9.6)	0.904
Missing	64 (1.4)	3 (1.1)	61 (1.4)	0.700
Type 1 Diabetes, *n* (%)	166 (3.5)	5 (1.8)	161 (3.6)	0.118
Biometric Factors, *n* (%)				
HbA1c Testing	4380 (92.4)	269 (97.8)	4111 (92.0)	<0.001
HbA1c < 8%	3835 (82.4)	210 (76.6)	3625 (82.7)	0.010
HbA1c > 9%	443 (9.3)	29 (10.5)	414 (9.3)	0.479
BP < 140/90 mmHg	3373 (71.3)	203 (73.8)	3170 (71.2)	0.349
Microalbumin Testing	3123 (65.8)	238 (86.6)	2885 (64.6)	<0.001
LDL Testing	3307 (69.7)	218 (79.3)	3089 (69.1)	<0.001
LDL < 100 mg/dL	2994 (71.0)	176 (70.1)	2818 (71.0)	0.750
BMI < 30 kg/m^2^	2185 (46.3)	129 (47.3)	2056 (46.3)	0.754
Diabetic Complications, *n* (%)				
Nephropathy	569 (12.6)	38 (13.8)	558 (12.5)	0.518
Peripheral Neuropathy	1811 (38.2)	99 (36.0)	1712 (38.3)	0.444

^†^ Includes self-pay or no listed insurance.

**Table 2:** Multiple logistic regression analysis of variables associated with completion of TRI. HbA1c, hemoglobin A1c; LDL, low-density lipoprotein.

Parameter	β	Standard Error	Wald χ^2^	OR	95% CI	*p*-Value
Male Sex (relative to female)	0.572	0.138	17.265	1.772	1.353–2.322	<0.001
Other Race/Ethnicity (relative to White) ^†^	0.351	0.150	5.474	1.420	1.059–1.905	0.019
HbA1c < 8%	−0.346	0.150	5.301	0.708	0.527–0.950	0.021
Completion of Biometric Testing						
HbA1c	0.577	0.441	1.711	1.781	0.750–4.229	0.191
Microalbumin	1.080	0.188	33.157	2.945	2.039–4.253	<0.001
LDL	0.259	0.161	2.594	1.295	0.945–1.775	0.107
Constant	−4.530	0.439	106.701	0.011		<0.001

^†^ Excluded from the analysis are 36 patients for whom race or ethnicity data was missing.

**Table 3:** Cost estimation for implementing remote DR screening based on time-driven activity-based costing and the personnel involved. TDABC, time-driven activity-based costing.

Modality	Required Personnel	Annual Salary (USD in Thousands)	Hours Worked	Wage Per Hour (USD)	Time Required (Hours)	TDABC (USD)
In-person Eye Examination	Ophthalmologist	299	1800	166	0.25	41.53
	Optometrist	127	1800	71	0.25	17.64
Remote Screening Examination	Medical Assistant	N/A	1800	17	0.20	3.40
Image Grading	Ophthalmologist	299	1800	166	0.03	4.98

## Data Availability

Due to the nature of this research, participants of this study did not agree for their data to be shared publicly, so supporting data are not available.

## Supplementary Materials

The following are available online at https://www.mdpi.com/article/10.3390/vision7030053/s1, Table S1: Sensitivity analysis using net present value for a TRI site.
