# Supplementary material for: Factors Associated with Utilization of Teleretinal Imaging in a Hospital-Based Primary Care Setting
Source: Vision (Basel). 2023 Aug 4;7(3):53. doi: 10.3390/vision7030053 (PMC10443374; doi:10.3390/vision7030053)
Supplement: Supplementary file 1 [file vision-07-00053-s001.zip › vision-2472873-supplementary.pdf]

Supplemental Table S1. Sensitivity analysis using net present value for a teleretinal imaging site.

	20 Screenings per Month	40 Screenings per Month	60 Screenings per Month
Year 1	($5311)	($2739)	$2405
Year 3	$4412	$12,127	$27,558
Year 5 †	$14,135	$26,903	$52,520

† Estimated service life of a hand-held fundus camera.
